# A control problem for a cross-diffusion system in a nonhomogeneous medium†

**DOI:** 10.1080/17513758.2013.836574

**Published:** 2013-09-18

**Authors:** Gabriela Marinoschi

**Affiliations:** a Institute of Mathematical Statistics and Applied Mathematics of the Romanian Academy, Bucharest, Romania; b Simion Stoilow Institute of Mathematics of the Romanian Academy, Research Group of the project PCCE 55/2008, Bucharest, Romania

**Keywords:** reaction–diffusion system, chemotaxis, perturbation method, boundary value problems for nonlinear parabolic PDE

## Abstract

We study a model, motivated by a bioremediation process, describing a cross-diffusion movement of a bacteria population *b* attracted by a chemoattractant signal *c*, in a nonhomogeneous stratified medium with *n* layers. We assume that this reaction–diffusion process is characterized by a low rate of degradation and a low diffusion coefficient of the chemoattractant, expressed in the model by a small parameter *∊*. The model consists of *n* systems of nonlinear parabolic equations with transmission conditions between layers. We prove a global-in-time solution for the asymptotic model setup with respect to the small parameter of the problem, for arbitrarily large initial data. Next, we deal with the control problem focusing mainly on the reduction of the chemoattractant concentration, by acting upon the initial distribution of the bacteria population *b*_0_. To this end, we prove the existence of a solution to the control problem and determine the optimality conditions.

*AMS Subject Classifications*: 35K57; 30E25; 35B20; 35K60

## 1. Introduction

Chemotaxis is the biological process of directed movement of cells in response to a chemical signal emitted by a substance or by another population, called chemoattractant, and play animportant role in the interaction of cells with their environment. Chemotaxis is a complex process which involves many aspects such as various species of cell populations *b* can interact; the movement can be either towards the higher concentration of the signal (positive chemotaxis) or away from it (negative chemotaxis); the chemical signal can be secreted by a population *b* itself, and not necessarily by an external source; the chemotactic process can lead to an aggregation of the attracted individuals and to a production of the chemoattractant, or contrary, it can determine the degradation of the chemoattractant. Consequently, the theoretical understanding of these processes determined a growing interest in their mathematical modelling, which at its turn raised challenging problems.

The origin of the fundamental model is given in the work of Patlak [24]. Later, Keller and Segel [18-20] introduced a similar model based on another assumptions. Since then, a rich mathematical literature on various versions of the model has been emerged, mainly focusing on the well-posedness of it and we refer the reader to a very comprehensive survey in [15].

In this paper, we shall denote the density of the cell population by *b* and the density of the population spreading the signal by *c*, both of them being functions of time *t*, and space variable *ξ*.

A chemotactic system with only one population of cells consists of two equations for *b* and *c* with initial and boundary conditions:


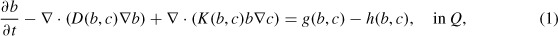



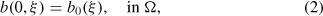



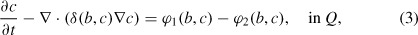



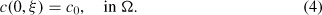


Here, the time runs in (0, *T*), *T* is finite, *ξ* ∊ Ω which is an open bounded subset of ℝ^*d*^ and *Q* = (0, *T*) × Ω. In these equations, *D*(*b, c*) and *δ*(*b, c*) represent the diffusion coefficients of the attracted population *b* and chemoattractant *c*, respectively; *g*(*b, c*) and *h*(*b, c*) are functions describing the rates of growth and death of *b*; and *φ*_1_ (*b, c*) and *φ*_2_ (*b, c*) stand for the production and degradation of the chemoattractant. Further, we denote the right-hand sides by *f* (*b, c*) = *g*(*b, c*) − *h*(*b, c*) and *φ*(*b, c*) = *φ*_1_ (*b, c*) − *φ*_2_ (*b, c*). They may depend on the space variable, too. The function *K* characterizes the chemotactic sensitivity and the cross-diffusion term in the first equation is indeed able to enforce the spontaneous emergence of structures provided that the process of chemotactic migration is accompanied by a production of the signal substance by the cells themselves [14]. Thus, the cross-diffusion term and the kinetic term *φ*(*b, c*) in the second equation can lead to the blow-up of the solution, even if the growth-death rate *f*(*b, c*) is zero and *D, δ, K* are constant. In the literature, the chemotactic system has been approached in simplified versions, able to avoid blow-up and to allow global solutions. We cite some more recent results.

In the 1-*D* case, it has been shown that blow-up does not occur [16] for *D* = 1, *K* constant and *δ* = *∊* small. When the space dimension *d* is greater or equal to two, the solutions generally exhibit blow-up, this being influenced by the model parameters and the characteristics of the initial data [13, 14, 25]. For example, in [17], a chemotaxis motion with constant diffusion coefficients is studied by using a nonlocal gradient sensing term to model the effective sampling radius of the species. In [9], Dyson *et al.* use a nonlocal term to model the species-induced production of the chemoattractant, *φ*_2_(*b, c, ξ*), in order to prevent blow-up in the *d-D* space, considering that the diffusion coefficients are constant. They prove the existence of solutions, which exist globally, and are *L*^∞^-bounded on finite time intervals. In [28], the system (1)–(4) is considered with *D*(*b, c*) = *δ*(*b, c*) = *K*(*b, c*) = *1, f* (*b, c*) = 0 and *φ*(*b, c*) = −*bc*, and it is shown that for arbitrarily large initial data, this problem admits at least one global weak solution for which there exists *T* > 0, such that (*b, c*) is bounded and smooth in (*T*, ∞) × Ω. The paper [8] deals with two types of reaction-diffusion systems, one arising in chemotaxis and the other in angiogenesis. The first refers to Equations (1)–(4) with *D*(*b, c*), *δ* (*b, c*) and *K*(*b, c*) constant, *f*(*b, c*) = 0, *φ*(*b, c*) = *b* − *αc* and the equation for *c* stationary, that is, — Δ*c* = *b* − *αc*. This equation is obtained as a case limit, for *∊* → 0, of Equation (3) in which *φ*_1_ (*b, c*) − *φ*_2_ (*b, c*) = (1/*∊*)(*b* − *c*), and the diffusion coefficient *δ* is of the order of 1/*∊*, with *∊* small. For this system (as well as for that of angiogenesis type), it is shown in [8] that when the *L*^*d*/2^ norm of initial data is small enough (for *d* ≥ 2), then there is a global (in time) weak solution that stays in all *L^p^* spaces with max{1; *d*/2 − 1} ≤ *p* < ∞. In [4], the same system but with *α* = 0 is studied in ℝ^2^ and a detailed proof of the existence of weak solutions below the critical mass, above which any solution blows up in finite time in the whole Euclidean space, is given [26]. The stability of the stationary solutions to a chemotaxis system was proved in [11] for *D* = 1, *δ*(*b, c*) = 0 and a general function *φ*(*b, c*). In [23], existence and uniqueness are studied for slow and singular fast diffusion of the cells in the case with a stationary equation for the chemoattractant.

Besides applications in biology, a chemotaxis model can apply in environment bioremediation in the cases when specific bacteria *b* are injected into a polluted medium (soil or water) with the purpose of cleaning it from an inside spread pollutant *c* [5, 10, 27].

Our study is motivated by an application to environment bioremediation and focuses on the case in which the kinetic term and the diffusion coefficient of the chemoattractant (pollutant) *c* have a weak influence on the flow, meaning that the rate of degradation of the chemoattractant is slow and it diffuses very little (or not at all, as in the case of oil polluting an environment).

Roughly speaking we shall start from a model reading as


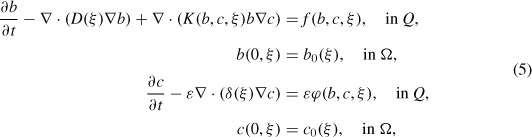


where *∊* is a small parameter in front of the diffusive and the kinetic terms for the chemoattractant. Such a model is obtained by making dimensionless Equations (1)–(4). Moreover, we assume that at the initial time *t* = 0 the chemoattractant concentration *c*_0_ is constant and that there exists a certain nonlinear dynamics growth-death of the cell population, indicated by *f*.

The model will be implemented into a nonhomogeneous medium, fact evidenced later by a particular space dependence of the coefficients. The aim is to investigate the way in which the solution depends on the small parameter *∊* and not to study the limit model when *∊* → 0. Accordingly, we shall not pass to the limit, but use a perturbation technique [7], by which the solution is expanded in series with respect to the powers of the small parameter, and retain the systems of *∊^m^*-order of approximation, obtained by equating the coefficients of *∊^m^* in Equations (5).

The main goal of the paper is to study the possibility of controlling the environment cleaning by acting upon the initial distribution of the bacteria. More exactly, the intention is to design the initial distribution of the bacteria such that the chemoattractant mean concentration should decrease under a certain critical value *c*_crt_ within a time period *T*.

A secondary task of the control problem is motivated by the fact that bioremediation can be problematic because sometimes it might become difficult to remove the microorganisms released into the environment in order to let them at acceptable levels in the soil [21]. Consequently, for enhancing a complete and efficient decontamination process and for avoiding higher costs of a further cleaning of the environment from the remained bacteria, we investigate by the same control problem whether a moderate bacteria proliferation, whose growth is limited by a prescribed density value *b_c_*, would be sufficient for achieving the main objective of reducing the pollutant concentration up to a value under the dangerous critical threshold *c*_crt_. To this end, the state system is studied in the framework of an appropriate functional setting and a global-in-time solution for the asymptotic system derived from Equations (5) is obtained. Then, the existence of at least a solution to the control problem is proved and the optimality conditions proving information about the initial distribution of the bacteria *b*_0_ are computed.

## 2. Statement of the model and the perturbation technique

We consider a nonhomogeneous 3D right cylinder domain


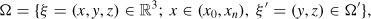


where the base Ω′ is an open bounded subset of ℝ^2^, with a boundary of class *C*^2^. The medium nonhomogeneity is modelled by a stratification of Ω in *n* parallel layers along the *Ox* axis, the separation of the layers being determined by the different values that movement parameters may have in each layer. More exactly, a stratification can be put into evidence when certain parameters do not depend on *x* (here *x* is the stratification variable) or are constant in each layer (*x*_*i*−1_, *x_i_*) but have different values from a layer to the other.

Therefore, the domain Ω consists of *n* subdomains Ω_*i*_, having the boundaries *∂*Ω_*i*_ = Γ_*i*−1_ ∪ Γ_*i*_ ∪ Γ_*i*_^lat^, *i* = 1, …, *n*, where Γ_*i*_^lat^ are the lateral boundary of Ω_*i*_ and Γ_*i*_ = {*x* = *x_i_*}, *i* = 0, …, *n*. The surfaces Γ_0_ and Γ_*n*_ are the external horizontal boundaries, while Γ_*i*_ with *i* = 1, …, *n* − 1 are the boundaries between layers. We denote


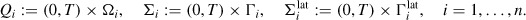


In each layer *i* the chemotaxis process is modelled by two equations, one for *b_i_* and the other for the chemoattractant *c_i_*. The interaction between the layers is established by transmission conditions for *b_i_*, that is, the continuity of the solutions and fluxes. We assume that the system is closed for *b_i_* and *c_i_*, namely the fluxes across the exterior frontiers are zero. The chemoattractant *c_i_* may display jumps at the interface between layers, because the *∊^m^*-approximation systems will not be anymore of diffusion type, as we shall see. Therefore, there is no need to specify these conditions.

The stratification is set by the values for *D_i_, δ_i_, c*_*i*,0_ assumed constant in each layer *i*, but different for two consecutive layers. Also, the expressions of the functions *f_i_, K_i_, φ_i_*, which are assumed not to depend explicitly on ξ, are different from one layer to another.

With these considerations, the dimensionless mathematical model for a chemotaxis movement in a nonhomogeneous stratified medium has *n* equations for the unknowns *b_i_* and *c_i_* (the density of the cell population and the chemoattractant concentration, respectively, in each layer *i* = 1, …, *n*) and reads [1]


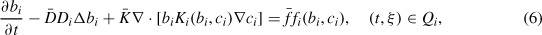



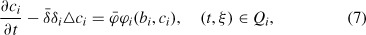



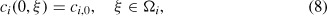



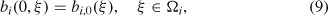


for all *i* = 1, …, *n*, where *c*_*i*,0_ (assumed constant) and *b*_*i*,0_ (ξ) are initial conditions for *c_i_* and *b_i_*. At the interface between two layers, we have the conditions


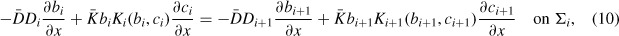



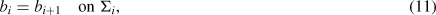


for *i* = 1, …, *n* − 1, and on the exterior horizontal and lateral boundaries, we set


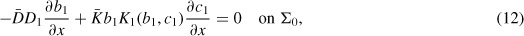



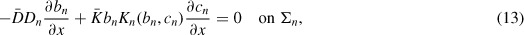



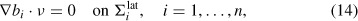



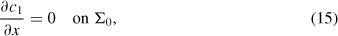



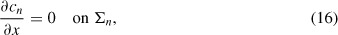



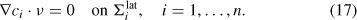


Here, *ν* is the unit outer normal to Γ_*i*_^lat^ and *∂*/*∂ ν* is the normal derivative.

Generally, *c_i_* and its normal derivative *δ_i_*(*∂c_i_*/*∂x*) can have a jump when crossing the internal boundaries Γ_*i*_. As we said, do not specify here these conditions because they will not intervene in the asymptotic model we shall study.

We stress that this model is presented in a dimensionless form and the constants *D, K, f, δ, φ*, are dimensionless parameters. They appear after the dimensionless procedure by which all dimensional variables and function are scaled by certain characteristic values indicated by the subscript *a* (e.g. *L_a_* for length, *T_a_* for time, *D_a_* for diffusion coefficient, etc.). We do not present the dimensionless computation, but we assert that by this procedure the dimensionless parameters are given by


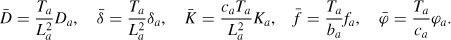


If by such a procedure the computed values *δ* and *φ* follow to have the same order of magnitude, much smaller than that of the other parameters, we set in Equations (6)–(17)


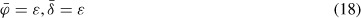


and consider the other dimensional parameters of order *O*(1) as *∊* → 0. We agree that this model characterizes the chemotactic process with a chemoattractant (e.g. oil in a bioremediation process) that diffuses very slow, and it is degraded with difficulty by the bacteria. This process happens in a different way in each layer, due to the nonhomogeneity modelled by the various functions *f_i_, K_i_, φ_i_*, the structure of the initial data and different diffusion coefficients.

### 2.1. Hypotheses

To approach system (6)–(17) we assume the following hypotheses for all *i* = 1, …, *n*:

(**i**_1_) *c*_*i*,0_ is constant, *c*_*i*,0_ ≥ 0, and there exist an *i* such that *c*_*i*,0_ > 0;
(**i**_2_) *b*_*i*,0_ ≥ 0, and there exist an *i* such that *b*_*i*,0_ > 0;
(**i**_3_) *D_i_* ≥ *D*_0_ > 0, *D*_0_ = min_*i*=1,*n*_ − *D_i_*;
(**i**_4_) (*r*_1_, *r*_2_) → *K_i_*(*r*_1_, *r*_2_) are of class *C*^1^ with respect to *r*_1_, |*K_i_*(*r*_1_, *r*_2_)|, |(*∂K_i_*/*∂r*_1_)(*r*_1_, *r*_2_)| are bounded, ∀*r*_1_, *r*_2_ ∊ ℝ;
(**i**_5_) (*r*_1_, *r*_2_) → *φ_i_* (*r*_1_, *r*_2_) are of class *C*^2^ with respect to *r*_1_ and |*φ_i_*(*r*_1_, *r*_2_)|, |(*∂φ_i_*/*∂r*_1_)(*r*_1_, *r*_2_)|, |(*∂*^2^*φ_i_*/*∂r*_1_^2^)(*r*_1_, *r*_2_)| are bounded.

We remark that equations with nonlinear terms *f_i_* do not generally admit global solutions in time [12], but by the perturbation technique we shall deduce a global solution for this asymptotic model, even under the assumption of a polynomial form for *f_i_*. Thus, for all *i* = 1, …, *n*, we assume


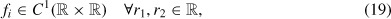



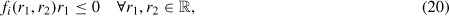



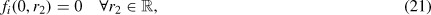



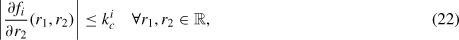



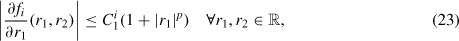


with *C*_1_*^i^, k^i^_c_* positive for *i* = 1, …, *n*, where


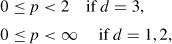


and *d* is the space dimension.

### 2.2. ∊^0^-order and ∊^1^-order approximations

We write the series expansions of all functions, with respect to the small parameter *∊* = φ = *δ*. Denoting generically *ϕ_i_*(*t, ξ*) and Φ_*i*_(*b_i_, c_i_*), we have


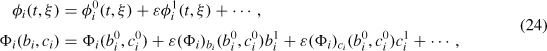


where *ϕ_i_^m^* corresponds to the *∊^m^*-approximation of *ϕ_i_* (*m* = 0, 1, …), and (Φ_*i*_)*_bi_*, (Φ_*i*_)*_ci_* represent the derivatives of Φ_*i*_ with respect to *b_i_* and *c_i_*.

We replace these series in the system (6)–(17) and by equating the coefficients of the *m*th powers of *∊*, we deduce the systems corresponding to *∊^m^*-order approximation.

The *∊*^0^-order approximation for *c_i_* reads


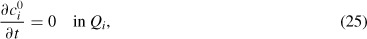



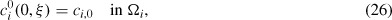


for each *i* = 1, …, *n*, implying that *c_i_*^0^ is a positive constant in each layer,


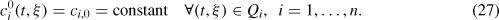


For *b*_*i*_^0^ we get


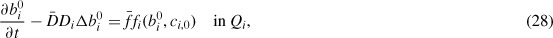



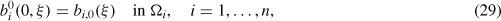



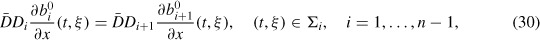



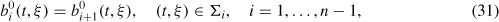



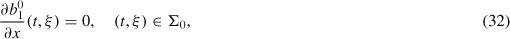



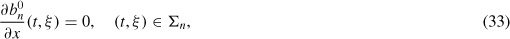



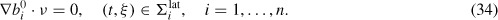


Further, identifying the coefficients of *∊*^1^ and using Equation (27), we obtain the system for the *∊*^1^-order approximation,


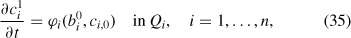



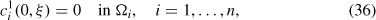



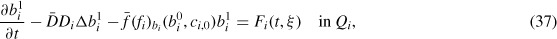



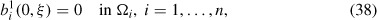



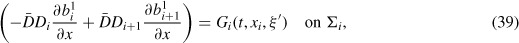



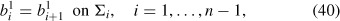



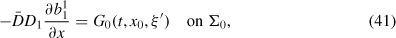



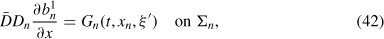



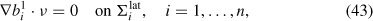


where we have denoted *ξ*′ ≔ (*y, z*) ∊ Ω′,


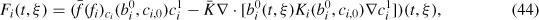


for *i* = 1, …, *n*,


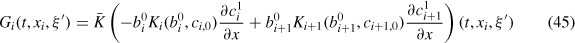


for *i* = 1, …, *n* − 1,


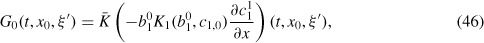



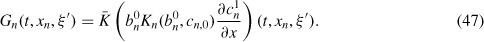


After solving the system for the *∊*^0^-order approximation, *c*_*i*_^1^ follows by Equations (35) and (36) and so the functions *F_i_*(*t, ξ*), *G_i_*(*t, x_i_, ξ′*) are known.

The next approximations (for *m* ≥ 2) lead to systems having similar forms as that for the *∊*^1^-order approximation, and so they pose the same mathematical problems. That is why we do no longer write them.

Definition 2.1 *We call an asymptotic solution to Equations* (6)–(17), *up to the order of approximation ∊*^2^, *a pair of functions* (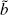
*_i_*, 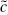
*_i_*), *i* = 1, …, *n*,

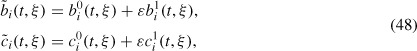

*where c_i_*^0^ *and c_i_*^1^ *are the solutions to the differential equations* (25) *and* (26) *and* (35) *and* (36), *respectively, and b*^0^*_i_ and b*^1^*_i_ are the solutions in the sense of distributions to Equations* (28)–(34) *and* (37)–(43).

We shall rigorously explain the definition of the solutions *b_i_*^0^ and *b*^1^_*i*_ in the next sections, in which the well-posedness of these systems will be studied.

### 2.3. The control problem

As we have already explained, the aim of the control problem is to provide information about the necessary initial density of bacteria, *b*_0_(ξ) = (*b*_*i*,0_(ξ))_*i*=1_, …,_*n*_, such that two objectives would be achieved. The main one is related to the environment cleaning that is, to force the decrease of the chemoattractant mean concentration under a critical threshold *c*_crt_. This means to minimize in the cost functional the mean positive part of the difference between the concentration 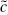
(*t, ξ*) = *c_i_*^0^(*t, ξ*) + *∊c*^1^_*i*_(*t, ξ*) given by Equation (48) and *c*_crt_. The second objective aims at realizing the necessary pollutant concentration decrease by a process limiting a too large proliferation of the bacteria. Consequently, in the cost functional we add a term expressing the restriction of the bacteria growth, by minimizing the mean positive part of the difference between *b*^0^_*i*_(*t, ξ*) and a constant prescribed value *b_c_*. We introduce this control problem to give a quick response (rather than an extremely accurate one) and to indicate a first decision for a process evolving in real time. That is why we accept that the consideration of the *∊*^0^-order approximation *b*^0^_*i*_ only, which is the dominant term in the asymptotic expansion of *b_i_*, is motivated. A more accurate computation, if necessary, may take into account the further approximations. Mathematically, we have to minimize the cost functional


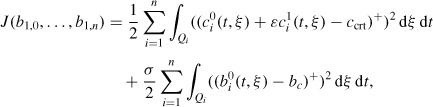


where the superscript '+’ means the positive part (i.e. φ+(ξ) = max{φ(ξ), 0}). A certain choice of the constant σ can induce a lower (or a higher) influence of the first term with respect to the second one in the cost functional. In particular, if the limiting condition of the proliferation of the bacteria is disregarded, the constant σ can be set zero.

Therefore, the control problem is


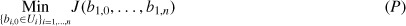


subject to Equations (28)–(34) and (35)–(36), where


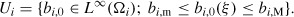


We recall that in our model, the initial distribution of the density of the bacteria may depend on the space variable ξ (i.e. *b*_0_(ξ) = (*b*_*i*,0_(ξ))_*i* = 1, …, *n*_) and only the chemoattractant initial concentration is considered constant (*c*_0_ = (*c*_*i*,0_)_*i* = 1, …, *n*_). A natural restriction for the initial densities of the bacteria *b*_*i*,0_ (*i* = 1, …, *n*), representing the controllers in the control problem, is to impose their boundedness. The minimum and maximum bounds, *b*_*i*,m_ and *b*_*i*,M_, are fixed numbers, 0 ≤ *b*_*i*,m_ < *b*_*i*,M_. However, as we see later, this restriction does not necessarily imply that the minimum of *J* must be reached at *b*_*i*,m_.

Finally, to be consistent with the requirement expressed by the first term in *J*, the maximum of the initial datum is chosen less than or equal to *b_s_*, the appropriate upper threshold envisaged for the solution *b*^0^_*i*_. In the case with σ = 0, the choice of *b*_*i*,M_ is free.

## 3. Well-posedness of the state system

In order to approach the control problem we need some information about the *∊*0 and *∊*^1^-state systems. We begin by a few preliminaries.

### 3.1. Preliminaries and functional setting

Since by our assumptions, *c*_*i*,0_ remains constant in each layer, one notes that in Equation (28) *f_i_* depends only on *b*^0^_*i*_ and so one can denote


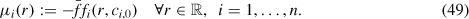


The assumptions (19)–(23) imply that the functions *μ_i_* ∊ *C*^1^(ℝ),


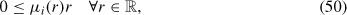



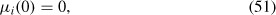



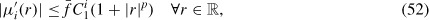


with *p* ∊ [0, 2) if *d* = 3 and *p* ≥ 0 if *d* = 1 or *d* = 2.

We rewrite the system (28)–(34) for the *∊*^0^-order approximation without indicating the superscript ‘0'


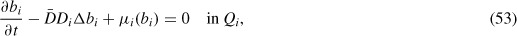



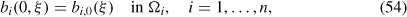



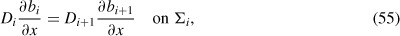



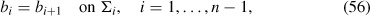



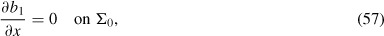



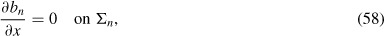



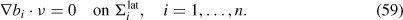


We introduce now the global functions defined in the following way (indicated by a generic notation *ϕ, ϕ*_0_):


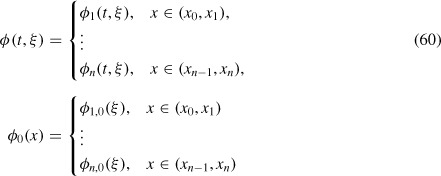


(for a function which is constant in each layer, as *c*_0_ or *D*),


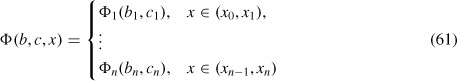


(for a function depending on *b* and *c*), where we recall that ξ = (*x, y, z*). We mention that we include the constant *D* in the definition of *D*(*x*).

We note that assumptions (50)–(52) and (**i**_1_)−(**i**_5_) imply similar properties for the functions defined before. Namely, we have


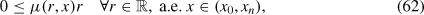



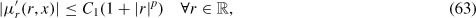



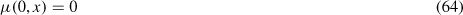


uniformly with respect to *x*, where *C*_1_ = *f* max_*i*=1,…, *n*_{*C*_1_^*i*^}, and *μ*′_*r*_ is the derivative with respect to *r*,


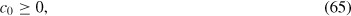



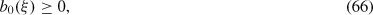



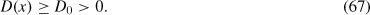


We recall that we consider *p* ∊ [0, 2) if *d* = 3 and *p* ≥ 0 if *d* = 1 or *d* = 2.

System (53)–(59) will be treated in the functional framework of the Sobolev space *V* = *H*^1^ (Ω) endowed with the standard norm ||*ψ*||*V* = (||*ψ*||^2^ + ||▽*ψ*||^2^)^1^/^2^, and its dual *V*′, with the pivot *H* = *L*^2^(Ω), such that *V* ⊂ *H* ⊂ *V*′. The value of *g* ∊ *V*′ at *ψ ∊ V* is


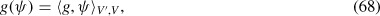


where 〈·, ·〉_*V*′,*V*_, represents the duality between *V*′ and *V*.

We specify that for the writing simplicity we shall denote the scalar product and the norm in *L*^2^(Ω) by (·, ·) and || · ||.

We define the operator *A*_0_ : *V* → *V*′ by


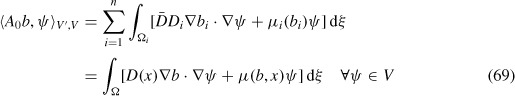


and restrict it to *L*^2^(Ω) by the operator *A* : *D*(*A*) ⊂ *L*^2^(Ω) → *L*^2^(Ω),


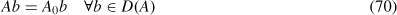


with *D*(*A*) = {*b* ∊ *V, Ab* ∊ *L*^2^(Ω)}.

Thus, the functional abstract setting of our problem is


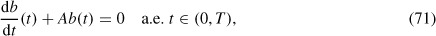



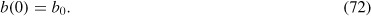


### 3.2. Existence for the ∊^0^-order approximation

The well-posedness for Equations (71) and (72) is concentrated in the following.

Theorem 3.1 *Assume conditions* (62)–(64), *and let b*_0_ ∊ *D*(*A*). *Then, problem* (71) *and* (72) *has a unique solution*

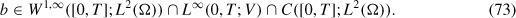

The solution satisfies the estimate

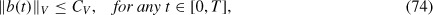

*where the constant C_V_ depends on the problem data. Moreover*,

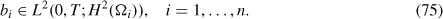

*Finally, let b*_0_ ≥ 0 *a.e. in* Ω. *Then, the solution b to Equations* (71) *and* (72) *satisfies*

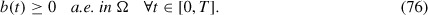

Proof The proof is split into two steps and is given in [1]. Further, we indicate the arguments. We assert by the properties (62)–(64) and by handling appropriate Sobolev inequalities that the operator *b* → *μ*(*b, x*) turns out to be locally Lipschitz from *V* to *L*^2^(Ω), uniformly in *x.* In the first step, according to the method presented in [3], we reduce the problem to a case with a globally Lipschitz operator, by approximating *μ*(·, *x*) by

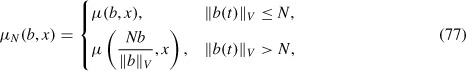

for each *N* natural. This truncated operator is Lipschitz from *V* to *L*^2^(Ω) for each *N* fixed, and we consider the approximating problem

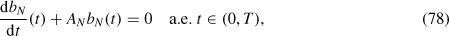

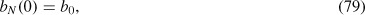

where *A_N_* is the operator *A* (defined by Equation (70)) in which *μ*(*b, x*) is replaced by *μ_N_*(*b, x*) and *D*(*A_N_*) = *D*(*A*).

For each *N*, it can be proved that *A_N_* is quasi-*m*-accretive on *L*^2^(Ω) and then, for *b*_0_ ∊ *D*(*A_N_*) = *D*(*A*), problem (78) and (79) has a unique strong solution

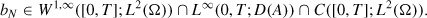

Some further necessary estimates are deduced for this solution, that is,

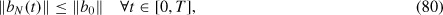

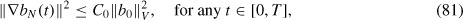

implying Equation (74), with *C*_0_ depending on fixed data of the problem (*p, T, D*_0_, *D*_∞_), and

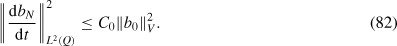

The proof of the *m*-accretiveness of *A_N_*, as well as all estimates written before involve some technical computations given in detail in [1].

The positiveness is proved on the basis of Stampacchia's lemma and Gronwall's lemma and show that the solution falls within the accepted physical domain of positive densities.

The regularity *H*^2^ (a.e. *t*) of the restriction of *b* to each layer *b_i_* comports a fine technique because it must adapt the known results [2, 6] for a domain with a regular boundary *∂*Ω to this transmission problem with *n* − 1 interfaces. These arguments are given in [1], too.

Finally, Equation (74) opens the ways to the second step by going back to Equations (71) and (72). In fact, if wechoose *R* = *C_V_* and *N* large enough, *N* > *R*, we get that *A_N_ b_N_* (*t*) = *Ab_N_*(*t*). Therefore, *b_N_* (*t*) with *N* large enough is the solution to problem (71) and (72), and further, it will be denoted by *b*.

For proving the uniqueness of the solution, we consider two solutions *b* and *b* corresponding to the same initial data *b*_0_. By the previous proof if

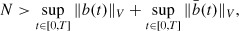

then we get *b*(*t*) = *b_N_*(*t*) and *b*(*t*) = *b_N_*(*t*), where *b_N_*(*t*) is the solution to Equation (78) and (79).

It is obvious that in each layer the functions *b_i_* (meaning in fact the *∊*^0^-approximation *b*^0^_*i*_) have the regularity induced by *b* restricted to *Q_i_*, for *i* = 1, …, *n*, that is,

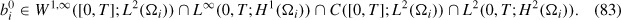

### 3.3. Existence for the ∊^1^-order approximation

The functions *c*^1^_*i*_ are computed by Equations (35) and (36),


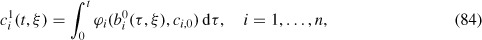


and by the boundedness hypothesis (**i**_5_) and Equations (83) and (75), we derive that


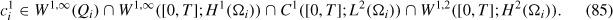


Existence for the solution to Equations (37)–(43) for the *∊*^1^-order approximation *b*^1^_*i*_(*t, ξ*) is studied by the Lions’ theorem for the time-dependent case [22] and the conclusions are [1] the following.

Theorem 3.2 *System* (37)–(43) *has a unique solution*

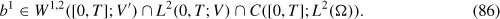

Corollary 3.3 *Problem* (6)–(17) *admits a unique asymptotic solution up to the order of approximation ∊*^2^,

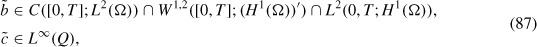

given by

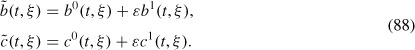

In particular, the restrictions of the solution to each layer have the properties


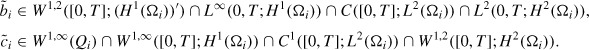


## 4. The control problem

Using the notation (60) we can rewrite the control problem (*P*) as


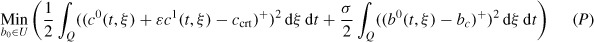


subject to Equations (53)–(59) (equivalently Equations (71) and (72)) and (84), where


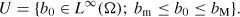


This means that *b*^0^ is the solution to the *∊*^0^-approximation, Equations (53)–(59), with the initial datum *b*^0^ and *c* is given by Equation (84). Here, *b_m_* = (*b*_*i*,m_)_*i*=1, …, *n*_ and *b_M_* = (*b*_*i*,M_)_*i* = 1, …,*n*_, and we note that *U* = Π_*i*=1_^*n*^ *U_i_*.

We recall that *c*^0^ (*t, ξ*) = *c*_0_ and is constant in all layers. Because in (*P*) only the *∊*^0^-order approximation for *b* and only the *∊*^1^-order approximation for *c* are involved, for the writing simplicity we shall indicate them by *b* and *c*, without superscripts.

### 4.1. Existence of the optimal control

Theorem 4.1 *Problem* (*P*) *has at least a solution.*

Proof Since *J* (*b*_0_) *≥* 0, its infimum exists and let us denote it by

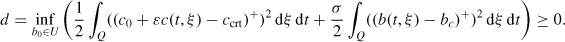

Let us take a minimizing sequence (*b^k^*_0_)_*k*≥1_, *b^k^*_0_ ∊ *U*, such that

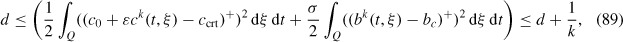

where *b^k^* = (*b^k^_i_*)_*i*=1, …, *n*_ is the solution to Equations (53)–(59) with initial datum *b^k^*_0_ and *c^k^* is given by Equation (84) corresponding to *b^k^*. We stress that the confusion between the sequence (*b^k^*)_*k*≥1_ and the *∊^k^*-order approximation should be avoided.

Since *b^k^*_0_ ∊ *U* we can select a subsequence (denoted still by *k*) such that

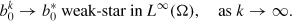

Then, the Cauchy problem (71) and (72)

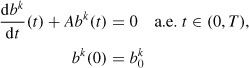

has, by Theorem 3.1, a unique solution *b^k^*, satisfying Equations (80)–(82) and (85). Therefore, selecting a subsequence we deduce that

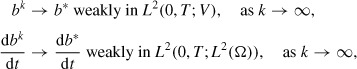

which implies by Lions' theorem (since *V* is compact in *L*^2^ (Ω)) that

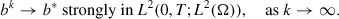

Then, *b^k^* → b* a.e. on *Q* and since *φ* is continuous it follows that *φ*(*b^k^*(*τ, ξ*), *c*_0_) → *φ*(b*(*τ, ξ*), *c*_0_) a.e. on *Q*, implying that (*c*_0_ + *∊c^k^* − *c*_crt_)^+^ → (*c*_0_ + ∊c* − *c*_crt_)^+^ a.e. in *Q*, as *k* → ∞. Moreover, *φ* is bounded by (**i**_5_), so by the Lebesgue dominated convergence theorem, we get by Equations (84) that

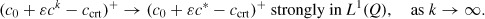

But, on the other hand, by Equation (89), the sequence (*c*_0_ + *∊c^k^* − *c*_crt_)^+^_*k*_ is bounded in *L*^2^ (*Q*), so that it converges weakly on a subsequence to a limit in *L*^2^ (*Q*), and in conclusion, by the limit uniqueness

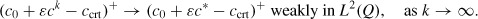

Since (*μ*(*b^k^*))_*k*_ lies in a bounded subset of *L*^2^ (*Q*) and *μ*(*b^k^*) → *μ*(b*) a.e. in *Q*, it follows that

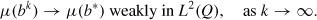

We also deduce by the Ascoli-Arzelà theorem that

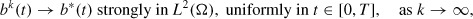

which implies that *b^k^*(0) = *b*_0_^*k*^ → b*_0_ as *k* → ∞. Moreover, b* is the solution to Equations (71) and (72) because, on the basis of the above convergencies, we can pass to the limit as *k* → ∞ in the weak form

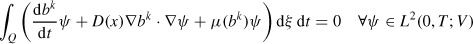

getting

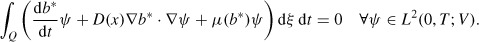

Finally, using the weakly lower semicontinuity property of the functions in Equation (89), we can pass to the limit and deduce

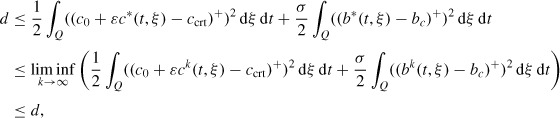

which shows that *J* (b*_0_) = *d*, hence (*P*) has at least a solution.

### 4.2. The system in variations and the dual system

Let (b*_0_, b*, c*) be an optimal pair in (*P*). The function b* is the solution to Equations (71) and (72) with the initial datum b*_0_ and c* is given by Equation (84) corresponding to b*,


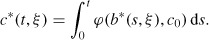


Let Δ > 0 and introduce *b*^λ^_0_ = b*_0_ + λ*w*, where


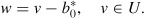


The corresponding solution to Equations (71) and (72) with the initial datum *b*^λ^_0_ is denoted by *b^λ^*, and we define


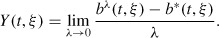


By a few computation, it can be shown that *Y* solves the system in variations (written for *Y_i_* in each layer)


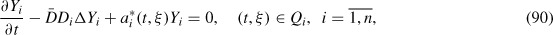



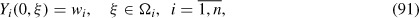



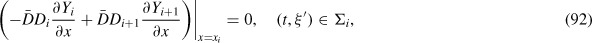



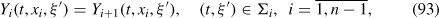



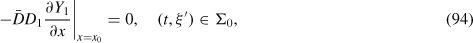



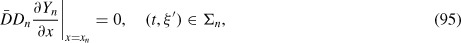



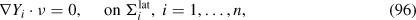


where *w_i_* = *v_i_* − b*_i,0_, with *v_i_* ∊ *U_i_* and a*_i_ : (0, *T*) × Ω*_i_* → ℝ,


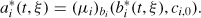


We see that the system in variations can be treated in the same manner as the system for the *∊*^1^-order approximation (being much simpler than it), and so it follows that it has a unique solution


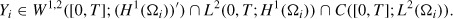


We introduce now the system for the dual variable *p*, written for each layer,


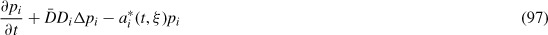



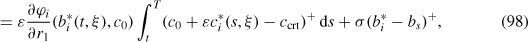



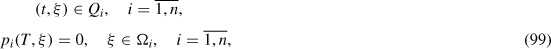



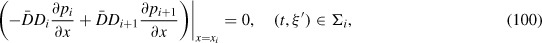



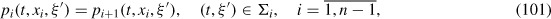



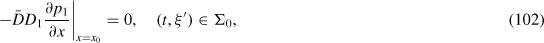



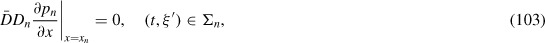



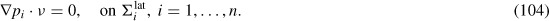


It is obvious that it has a unique solution


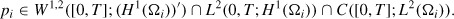


### 4.3. Optimality conditions

Proposition 4.2 *Let* (b*_0_, b*) *be optimal in* (*P*). *Then, the optimality conditions read*

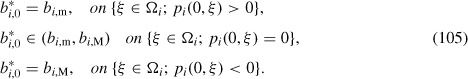

Proof We write that (b*_0_, b*) is optimal, that is,

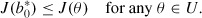

We take *θ* = *b^Δ^* and have

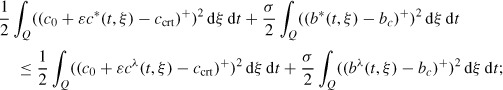

whence, passing to the right-hand side, performing some computations, dividing by *λ* and letting *λ* to go to 0, we get

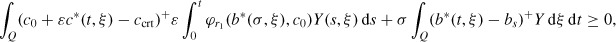

where *φ*_*r*1_ generically indicates ∂*φ_i_*/∂*r*_1_ in each layer. This can still be written, after changing the order of integrations, as

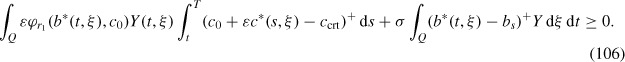

Then, we test Equation (90) by *pi* and integrate over (0, *T*), using the initial data and boundary conditions. We obtain writing globally

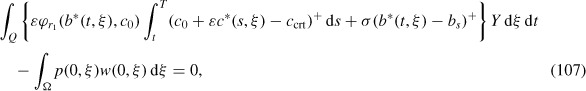

where we recall that *w*(ξ) = *v*(ξ) − b*_0_(ξ). Combining Equations (106) and (107) we finally deduce

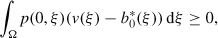

which can be still written

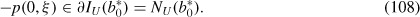

In this relation, ∂*I_U_* (b*_0_) is the subdifferential at b*_0_ of the indicator set of *U* and *N_U_*(b*_0_) is the normal cone to *U* at b*_0_. In conclusion, we obtain relations (105) as claimed.

We recall that *b_m_* and *b_M_* represent a sequence of minimum and maximum values assigned for each layer *i.* Equivalently, the previous relation −*p*(0, ξ) ∊ *N_U_*(b*_0_) means that −*p_i_*(0, ξ) ∊ *N_U_i__* (b*_0_) and so the relations given by relations (105) follow.

We observe that the minimization problem admits at least a solution which can be realized not necessarily at *b*_0_ = *b_m_*, but in a way expressed by relations (105). We conclude that the controller b*_0_ (= b*_0_) may take either the value *b*_*i*,m_ or *b*_*i*,M_ on the subsets of Ω_*i*_, where *p_i_* (0, ξ) has positive or negative values. In some layers, b*_0_ may reach also the value 0 (if e.g.*p_i_* (0, *ξ*) > 0 in Ω_*i*_), because *b*_*i*,m_ was allowed to be nonnegative. This means that in some situations it might be possible not to place bacteria in some layers at the initial time. Finally, we assert that it is difficult to say if the system (98)–(104) can have a null solution *p_i_*(0, ξ) on a subset of Ω_*i*_, but this analysis is beyond the scope of this paper. Therefore, we cannot exclude that in some circumstances it may happen that the minimum be reached for whatever *b*_*i*,0_ ∊ (*b_i,m_*, *b*_*i*,m_).

## 5. Numerical results

Some graphics revealing the feature of the process with respect to the change of *b*_0_ are presented. The simulations are made with Comsol Multiphysics (FLN License 1025226) for the system (6)–(17) in a 1D domain with the same values *D, δ, χ, c*_0_ in all layers (in order to compare how results change for various *b*_0_) with the following dimensionless data:


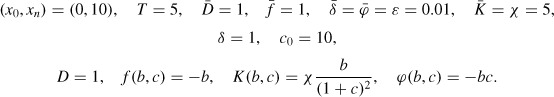


**Figure 1.**
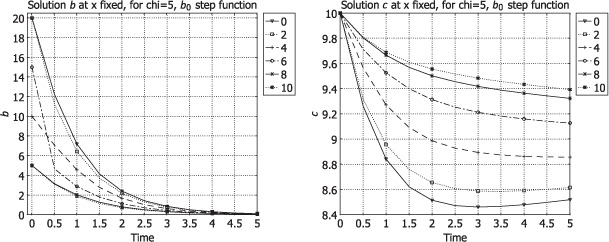
Solutions *b*(*t, ξ*) (left) and *c*(*t, ξ*) (right) for *b*_0_(*x*) given by (109).

**Figure 2.**
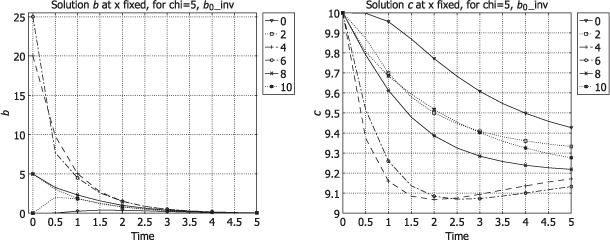
Solutions *b*(*t, ξ*) (left) and *c*(*t, ξ*) (right) for *b*_0inv_ (*x*) given by (110).

The figures represent the values of *b* and *c* along time, drawn at *x* fixed (0, 2, 4, 6, 8, 10) corresponding to two values of the initial datum *b*_0_. In Figure 1, these graphics are plotted for *b*_0_, the step function


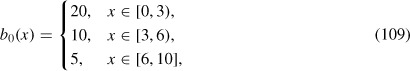


while in Figure 2 the graphics are displayed for


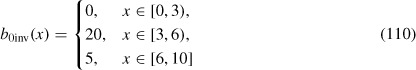


In Figure 1 (right), we see that the values of *c* increase from *x* = 0 to *x* = 10. The medium cleaning is more efficient beginning with the first layers. In Figure 2 (right), the medium cleaning happens in a different way, being less efficient in the first layers (*x* ∊ [0, 2]) where the values of *c* remain high, but more efficient in the medium layers (*x* ∊ [4, 6]).

## 6. Conclusion

We address a control problem related to a chemotactic motion of a bacteria towards a pollutant chemoattractant in a stratified medium. The control problem is focused on the reduction of the chemoattractant concentration, by acting upon the initial distribution of the bacteria population, *b*_0_. We prove that the control problem has at least a solution and provide the structure of the controller *b*_0_ in each layer.
